# Changes in dementia treatment patterns associated with changes in the National Policy in South Korea among patients with newly diagnosed Alzheimer’s disease between 2011 and 2017: results from the multicenter, retrospective CAPTAIN study

**DOI:** 10.1186/s12889-024-17671-2

**Published:** 2024-01-12

**Authors:** Young Jin Kim, Ki-Youn So, Hyo Min Lee, Changtae Hahn, Seung-Hoon Song, Yong-Seok Lee, Sang Woo Kim, Heui Cheun Park, Jaehyung Ryu, Jung Seok Lee, Min Ju Kang, JinRan Kim, Yoona Lee, Jun Hong Lee

**Affiliations:** 1 Sungae Hospital, Seoul, South Korea; 2 Namwon Medical Center, Namwon-si, Jeollabuk-do South Korea; 3 Daegu Fatima Hospital, Daegu, South Korea; 4 Daejeon St. Mary’s Hospital, Daejeon, South Korea; 5 Baekje General Hospital, Nonsan-si, Chungcheongnam-do South Korea; 6 Seoul Metropolitan Government-Seoul National University Boramae Medical Center, Seoul, South Korea; 7 Busan St. Mary’s Hospital, Busan, South Korea; 8 Andong Medical Group Hospital, Andong-si, Gyeongsangbuk-do South Korea; 9 Yeosu Chonnam Hospital, Yeosu-si, Jeollanam-do South Korea; 10 Jeju National University Hospital, Jeju-si, Jeju-do South Korea; 11 Veterans Healthcare Medical Center, Seoul, South Korea; 12 Eisai Korea Inc, Seoul, South Korea; 13 National Health Insurance Service Ilsan Hospital, Goyang-si, Gyeonggi-do South Korea

**Keywords:** Dementia, Alzheimer’s disease, Treatment pattern, Medication persistence, Electronic medical records, National policy, Long-term care insurance, LTCI

## Abstract

**Background:**

The South Korean government has been actively involved in plans to combat dementia, implementing a series of national strategies and plans since 2008. In July 2014, eligibility for mandatory long-term care insurance (LTCI) was extended to people with dementia enabling access to appropriate long-term care including the cognitive function training program and home nursing service. This study aimed to investigate changes in treatment patterns for Alzheimer’s disease (AD) between July 2011 and June 2017 which spanned the 2014 revision.

**Methods:**

This multicenter, retrospective, observational study of patients with newly diagnosed AD analyzed electronic medical records from 17 general hospitals across South Korea. Based on their time of AD diagnosis, subjects were categorized into Cohort 1 (1 July 2011 to 30 June 2014) and Cohort 2 (1 July 2014 to 30 June 2017).

**Results:**

Subjects (*N*=3,997) divided into Cohorts 1 (*n*=1,998) and 2 (*n*=1,999), were mostly female (66.4%) with a mean age of 84.4 years. Cohort 1 subjects were significantly older (*P*<0.0001) and had a lower number of comorbidities (*P*=0.002) compared with Cohort 2. Mean Mini-Mental State Examination (MMSE) scores in Cohorts 1 and 2 at the time of AD diagnosis or start of initial treatment were 16.9 and 17.1, respectively (*P*=0.2790). At 1 year, mean MMSE scores in Cohorts 1 and 2 increased to 17.9 and 17.4, respectively (*P*=0.1524). Donepezil was the most frequently administered medication overall (75.0%), with comparable rates between cohorts. Rates of medication persistence were ≥98% for acetylcholinesterase inhibitor or memantine therapy. Discontinuation and switch treatment rates were significantly lower (49.7% *vs.* 58.0%; *P*<0.0001), and mean duration of initial treatment significantly longer, in Cohort 2 *vs.* 1 (349.3 *vs*. 300.2 days; *P*<0.0001).

**Conclusions:**

Comparison of cohorts before and after revision of the national LTCI system for dementia patients found no significant difference in mean MMSE scores at the time of AD diagnosis or start of initial treatment. The reduction in the proportion of patients who discontinued or changed their initial treatment, and the significant increase in mean duration of treatment, were observed following revision of the LTCI policy which enabled increased patient access to long-term care.

**Supplementary Information:**

The online version contains supplementary material available at 10.1186/s12889-024-17671-2.

## Background

The number of people worldwide living with dementia in 2020 was more than 55 million people and numbers are expected to increase to 78 million in 2030 and 139 million in 2050 [1]. In South Korea (henceforth Korea), analysis of big data from the National Health Insurance Service (NHIS) for dementia and hospital utilization for dementia show that the prevalence of dementia has increased significantly in recent years, notably among the elderly population (aged ≥65 years) [2–4]. In 2021, the prevalence of dementia in Korea was estimated to be more than 786,000 with numbers expected to continue to rise over the next two decades or more [5]. The health-economic burden of dementia in Korea is substantial and was estimated at US$6,957 per capita, with indirect costs accounting for 48.0% of the total burden, mainly from loss of productivity for family members and caregivers [6]. The total annual national dementia management cost for dementia patients in 2021 (approx. US$138 billion) accounted for about 0.9% of Korea’s GDP and, during a 6-year period from 2017, the cost increased by 31.9% [7].

In Korea, AD medication is available from hospitals and clinics. Data from 2021 for people with dementia in Korea (approx. 1.66 million), show that most treatments received were as outpatients (52.3%), followed by from a pharmacy (35.4%) or as inpatients (12.3%). Many people with dementia (*n* = 382,155) accessed long-term care insurance services for the elderly in 2021, with over two-thirds choosing to receive care at home (67.5%) rather than in care facilities [7].

The Korean government has been actively involved in plans to combat dementia, implementing a series of national strategies and plans, beginning in 2008 when the first national dementia plan was announced. Both the first and second national dementia plans, the latter being announced in 2012, focused primarily on promoting early detection and diagnosis of dementia by healthcare providers. The Dementia Management Act of 2012 established a statutory basis for the organization of national dementia plans. The third national dementia plan, released in 2016, focused on the community-based prevention and management of dementia and the fourth, released in 2020, deals with the prevention, early detection, and early post-diagnosis management of Alzheimer’s disease (AD) [8, 9].

Mandatory long-term care insurance (LTCI) was introduced in Korea in 2008 and eligibility was extended in July 2014 to people with dementia (including mild dementia). Prior to the 2014 revision, people with cognitive disorders but without severe physical disability were not eligible for LTCI [8, 10]. The revision enabled access to appropriate long-term care for many dementia patients (including mild AD patients) and their families including the cognitive function training program and home nursing services [11]. National policies continue to play a vital role in dementia care for the elderly, especially those with low income. These policies are essential for supporting the treatment of dementia including medications for AD and dementia.

This study aimed to investigate changes in treatment patterns for AD and assessed their effectiveness during two consecutive 3-year periods (July 2011 – June 2014 and July 2014 – June 2017) which spanned revision of the LTCI system regarding eligibility for dementia patients, in July 2014.

## Methods

The multicenter, retrospective, observational CAPTAIN (Change of treatment patterns for newly diagnosed Alzheimer’s Disease Patients According to Korean National Policy [Long Term Care Insurance] for dementia) study of patients with newly diagnosed AD analyzed electronic medical records (EMRs) from 17 general hospitals across Korea between July 2011 and June 2017. A complete list of all study sites and corresponding Institutional Review Boards (IRBs) that reviewed and approved the study protocol is provided in Supplementary Table 1. Subjects were categorized into two cohorts based on the time of AD diagnosis: from 1 July 2011 to 30 June 2014 (Cohort 1) and from 1 July 2014 to 30 June 2017 (Cohort 2).

### Variables

Data retrieved from patient EMRs included age, highest attained educational level, past medical history including comorbidities defined by MedDRA v24.1 System Organ Class (SOC) and Preferred Term (PT), AD-related medication history, Mini-Mental State Examination (MMSE) score [12], Clinical Dementia Rating (CDR) [13], and Global Deterioration Scale (GDS) [14].

### Inclusion and exclusion criteria

Inclusion criteria were patients who were newly diagnosed with AD between 1 July 2011 and 30 June 2017, attended a general hospital as an outpatient, and started acetylcholinesterase inhibitor (AChEI) or memantine administration during this period. Patients were required to have a verifiable MMSE score within 6 months prior to AD diagnosis or the start of initial treatment.

Exclusion criteria were patients with no records available for MMSE, CDR, and/or GDS between 1 July 2011 and 30 June 2017, and/or with a medication history of AChEI or memantine treatment prior to AD diagnosis.

### Objectives

The primary objective of this study was to compare MMSE scores between cohorts at the time of AD diagnosis or start of initial treatment. Secondary objectives were comparisons between cohorts of changes in MMSE scores after 1 year’s treatment, initial treatment medication and reasons for the discontinuation or change (add-on, switching) of treatment, and time from initial treatment initiation to diagnosis of depression or prescription of antidepressants.

### Statistical analyses

Continuous variables were summarized by mean, standard deviation (SD), median and range; and categorical variables by number and percentage. Statistical comparisons were made using Wilcoxon rank-sum, Chi-square or Fisher's exact tests except for Kaplan-Meier analyses which used log-rank tests. The significance level was set at 0.05 (two sided). All statistical analyses were conducted using SAS version 9.4.

## Results

In total, 3,997 subjects were enrolled in the study and there were no exclusions. Based on their time of diagnosis, subjects were divided into Cohort 1 (July 2011 – June 2014; *n* = 1,998) and Cohort 2 (July 2014 – June 2017; *n* = 1,999). Subjects were mostly female (66.4%) with a mean age of 84.4 years. Subjects in Cohort 1 were significantly older than those in Cohort 2 (mean age 84.9 *vs* 84.0 years; *P* < 0.0001). By age category, Cohort 1 had a lower proportion of subjects in the ≥70 to <80 years (19.3% *vs* 22.4%) and ≥80 to <90 years (46.2% *vs* 51.6%) age groups, but a higher proportion of subjects in the ≥90 years age group (30.2% *vs* 22.3%). The highest educational level attained was significantly different between cohorts (*P* < 0.0001). Approximately three quarters of subjects (75.7%) had one or more comorbidities. By PT, the most common comorbidities were hypertension (45.5%, *n* = 1,817) followed by diabetes mellitus (20.5%, *n* =820) and hyperlipidemia (8.8% *n* = 350). Cohort 1 had a lower proportion of subjects with ≥1 comorbidity compared with Cohort 2 (73.6% *vs* 77.8%; *P* = 0.0019). Cohort 1 had a lower prevalence of depression (11.8% *vs* 14.0%; *P* = 0.004), diabetes mellitus (19.1% vs. 21.9%;* P* = 0.0403) and hypertension (43.8% vs. 47.1%; *P* = 0.0289) compared with Cohort 1; and stroke was more common in Cohort 1 (24.5% *vs* 21.0%; *P* = 0.0137) (Table 1).

**Table 1:** Demographics and baseline characteristics

	**Cohort 1 (*n* = 1,998)**	**Cohort 2 (*n* = 1,999)**	**Total (*N* = 3,997)**	*P* value*: Cohort 1 *vs* 2
Sex: male/female, n (%)	657 (32.9)/ 1,341 (67.1)	685 (34.3)/ 1,314 (65.7)	1,342 (33.6)/ 2,655 (66.4)	0.3542
Age (years), Mean ± SD	84.9 ± 8.6	84.0 ± 7.5	84.4 ± 8.0	<0.0001
Age range (years)				
<40	1 (0.1)	0 (0.0)	1 (0.0)	<0.0001
≥40 to <50	5 (0.3)	0 (0.0)	5 (0.1)	
≥50 to <60	10 (0.5)	5 (0.3)	15 (0.4)	
≥60 to <70	71 (3.6)	70 (3.5)	141 (3.5)	
≥70 to <80	385 (19.3)	447 (22.4)	832 (20.8)	
≥80 to <90	923 (46.2)	1,031 (51.6)	1,954 (48.9)	
≥90	603 (30.2)	446 (22.3)	1,049 (26.2)	
Highest educational level				
No formal school education	383 (19.2)	471 (23.6)	854 (21.4)	<0.0001
Elementary school or below	544 (27.2)	600 (30.0)	1,144 (28.6)	
Middle school	102 (5.1)	133 (6.7)	235 (5.9)	
High school	111 (5.6)	147 (7.4)	258 (6.5)	
College/graduate school	58 (2.9)	80 (4.0)	138 (3.5)	
Unknown	800 (40.0)	568 (28.4)	1,368 (34.2)	
Past medical history				
Depression, n (%)	235 (11.8)	280 (14.01)	515 (12.9)	0.0040
Diabetes mellitus	382 (19.1)	438 (21.9)	820 (20.5)	0.0403
Hypertension	876 (43.8)	941 (47.1)	1,817 (45.5)	0.0289
Stroke, n (%)	490 (24.5)	419 (21.0)	909 (22.7)	0.0137
≥1 comorbidity, n (%)	1,470 (73.6)	1,555 (77.8)	3,025 (75.7)	0.0019

^*^Chi-square tests except Wilcoxon rank-sum test for mean age

Mean ± SD MMSE scores in Cohorts 1 and 2 at the time of AD diagnosis or start of initial treatment were 16.9 ± 6.1 and 17.1 ± 5.8, respectively (*P* = 0.2790). At 1 year, mean ± SD MMSE scores in Cohort 1 (*n* = 588) and Cohort 2 (*n* = 707) were 17.9 ± 6.1 and 17.4 ± 5.5, respectively. Differences in 1-year MMSE between cohorts were not significantly different (*P* = 0.1524). Mean ± SD change in MMSE score from treatment start to end of 1 year's treatment was +0.2 ± 3.6 in Cohort 1 (*n* = 588) and –0.2 ± 3.6 in Cohort 2 (*n* = 707). These differences were not statistically significant (*P* = 0.0711). In subjects stratified by disease severity at baseline [baseline MMSE score: 30-27 (normal), 26-21 (mild), 20-10 (moderate), <10 (severe)], there was a significant difference between cohort subgroups in change in MMSE at 1 year in subjects with mild disease (*P* = 0.0021), but not in subjects with normal, moderate or severe disease status (Supplementary Table 2).

Initial medications administered to AD patients differed significantly between cohorts (*P* < 0.0001). Donepezil monotherapy was the most administered medication overall (75.0%) and the administration rate in Cohort 1 was higher in Cohort 2 (77.1% and 72.9%, respectively). Rivastigmine was more commonly administered to patients in Cohort 1 (12.5% *vs.* 9.0%) while galantamine (6.81% *vs.* 10.91%) and memantine (3.6% *vs.* 3.8%) were more frequently administered to Cohort 2 patients. Combination donepezil + memantine was only administered to Cohort 2 subjects (3.4%) (Table 2). In a subgroup analysis (by 12-month period) of each cohort, donepezil was consistently the most common medication administered with some variation between the 12-monthly periods analyzed. Combination donepezil + memantine was most frequently administered during July 2014–June 2015 (Cohort 2-1) (Supplementary Table 3).

**Table 2:** Initial medications administered

	**Cohort 1 (*n* = 1,998)**	**Cohort 2 (*n* = 1,999)**	**Total (N = 3,997)**	*P* value*: Cohort 1 *vs* 2
	n (%)			
Donepezil	1,541 (77.1)	1,457 (72.9)	2,998 (75.0)	<0.0001
Rivastigmine	250 (12.5)	180 (9.0)	430 (10.8)	
Galantamine	136 (6.8)	218 (10.9)	354 (8.9)	
Memantine	71 (3.6)	76 (3.8)	147 (3.7)	
Combination donepezil + memantine	0 (0.0)	68 (3.4)	68 (1.7)	

^*^Chi-square test

Medication persistence, defined as the proportion of time during the prescribed duration for which patients continued treatment, was high (≥98%) for donepezil, galantamine, rivastigmine and memantine (Table 3). Mean medication persistence was significantly higher in Cohort 1 *vs* 2 for donepezil (98.7 *vs.* 98.4; *P* = 0.0001) and memantine (98.8 *vs.*98.7; *P* = 0.0339). In subjects stratified by disease severity at baseline, medication persistence for ChEIs or memantine was significantly different in mild (galantamine: *P* = 0.0285), moderate (donepezil: *P* = 0.0023; memantine: *P* = 0.0230), and severe (donepezil: *P* = 0.0424) AD subgroups (Supplementary Table 2).

**Table 3:** Medication persistence^a^ (%)

		**Cohort 1**	**Cohort 2**	**Total**	***P* value†: Cohort 1 *vs* 2**
Donepezil	n	1,514	1,473	2,987	
	Mean ± SD	98.7 ± 7.3	98.4 ± 6.8	98.57 ± 7.0	0.0001
Rivastigmine	n	249	178	427	
	Mean ± SD	98.3 ± 10.6	99.8 ± 2.8	98.9 ± 8.3	0.0500
Galantamine	n	136	215	351	
	Mean ± SD	98.7 ± 5.9	99.5 ± 3.8	99.2 ± 4.7	0.0785
Memantine	n	71	142	213	
	Mean ± SD	98.8 ± 7.2	98.7 ± 6.8	98.7 ± 6.9	0.0339

^a^Medication persistence is defined as the proportion of time during the prescribed duration for which patients continued treatment, calculated as:

$$\text{Medication Possession Ratio (MPR)} = \frac{\text{Actual number of days of taking medication*}}{\text{Planned number of days of taking medication**}} \times 100$$

*Treatment end date (end date of administration or end date of follow-up specified in the electronic medical record) - treatment start date (start date of administration)

**Number of prescribed days X times of prescription (in case the number of prescribed days were different, each number of prescribed days was added)

**†**Wilcoxon rank-sum test

Overall, the mean ± SD time from AD diagnosis to the start of initial therapy was 8.3 ± 39.6 days. Time to the start of therapy was significantly shorter in Cohort 1 (7.8 ± 41.0 days) compared with Cohort 2 (8.8 ± 38.2 days) (*P* = 0.0007). In subjects stratified by disease severity at treatment start, this difference was statistically significant in patients in the mild (*P* = 0.0427) and moderate (*P* = 0.0034) AD subgroups (Supplementary Table 2).

**Fig. 1:**
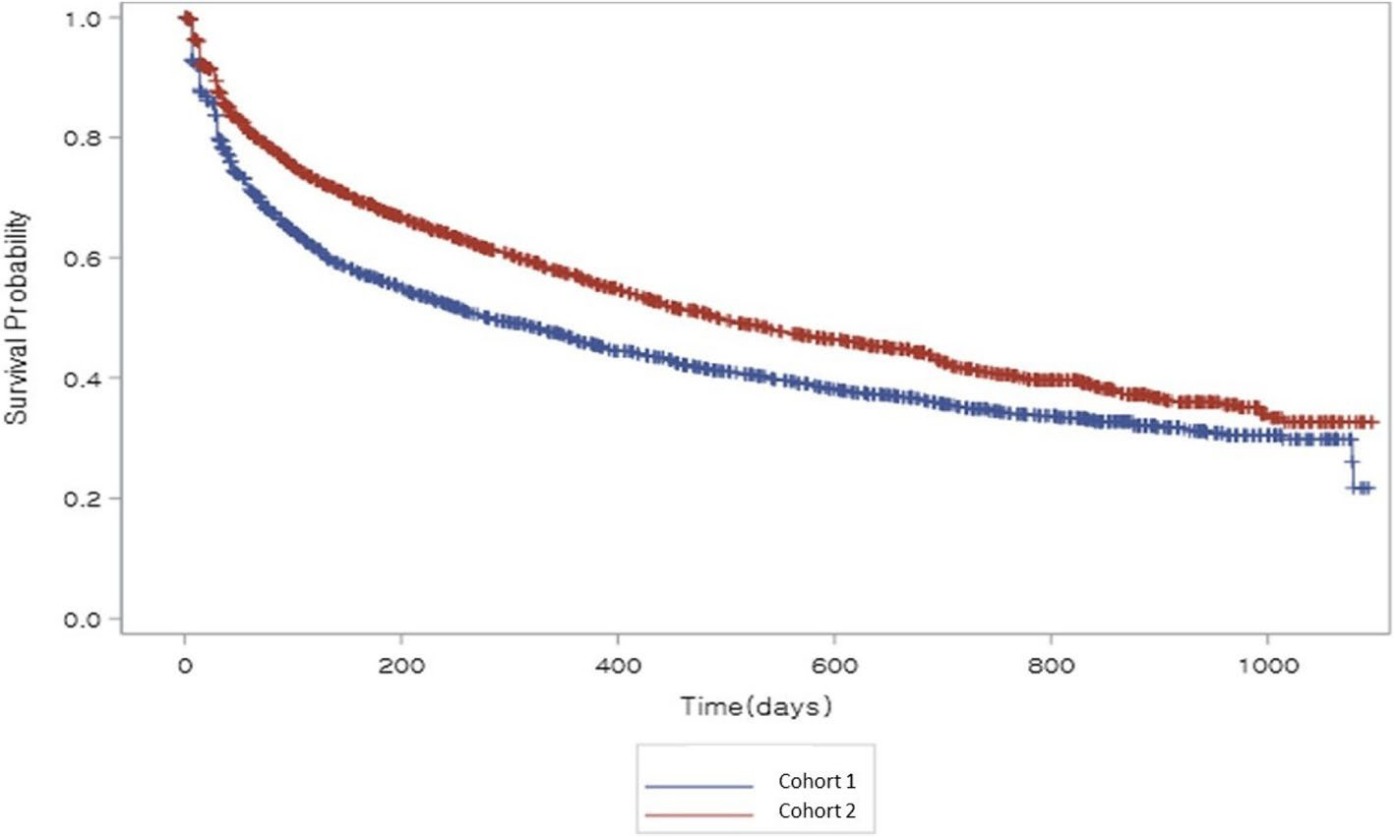
Kaplan-Meier analysis of initial treatment duration in subjects who discontinued or changed their initial treatment

Discontinuation and adjustment of initial treatment rates were significantly lower in Cohort 2 *vs.* Cohort 1 (49.7% *vs.* 58.0%; *P* < 0.0001). In subjects stratified by disease severity at baseline, this difference was statistically significant in the moderate AD subgroup (*P* < 0.0001) (Supplementary Table 2). For subjects who discontinued or changed their initial treatment, the mean ± SD overall duration of initial treatment was 324.8 ± 315.0 days. Kaplan-Meier analysis of initial treatment duration in Cohorts 1 and 2 who discontinued or changed their initial treatment is shown in Fig. 1. Mean duration of initial treatment was significantly longer in Cohort 2 (349.8 ± 316.1 days) than Cohort 1 (300.2 ± 312.0 days) (Log-rank test *P* < 0.0001). In subjects stratified by disease severity at treatment start, statistically significant differences were observed in the mild (*P =*0.0317), moderate (*P* < 0.0001), and severe (*P =*0.0286) AD subgroups (Supplementary Table 2).

Treatment interruption/discontinuation occurred in 2,190 subjects: 1,159 subjects in Cohort 1 (52.9%) and 1031 in Cohort 2 (47.1%). Overall, 1,587 subjects were lost to follow-up (39.7% of all subjects) and included 901 (45.1%) and 686 (34.3%) in Cohorts 1 and 2, respectively. The most frequent reason for discontinuation or change of initial treatment was lack of effectiveness (8.1%*vs* 11.1%, respectively), followed by adverse effects (2.3% *vs* 3.2%) and death (0.3% *vs* 0.7%) (Table 4). In subgroup analysis (by 12-month periods) of each cohort, interruption/discontinuation due to lack of effectiveness was higher during the first 12 months although numbers of subjects in each subgroup are low (Supplementary Table 4).

**Table 4:** Reasons for discontinuation or change of initial treatment in Cohorts 1 and 2

	**Cohort 1 (*n* = 1,998)**	**Cohort 2 (*n* = 1,999)**	**Total (*N* = 3,997)**
	n (%)		
Lost to follow-up	901 (45.1)	686 (34.3)	1,587 (39.7)
Lack of effectiveness	161 (8.1)	222 (11.1)	383 (9.6)
Adverse effects	45 (2.3)	63 (3.2)	108 (2.7)
Death	6 (0.3)	13 (0.7)	19 (0.5)
Economic burden	1 (0.1)	2 (0.1)	3 (0.1)
Symptom improvement	1 (0.1)	0 (0.0)	1 (0.0)
Other	44 (2.2)	45 (2.3)	89 (2.2)
Total	1,159 (58.0)	1,031 (51.6)	2,190 (54.8)

Percentages shown are for the proportion of subjects in each cohort

Overall, 136 patients added therapy due to lack of effectiveness of initial treatment medication: 29 subjects in Cohort 1 and 107 in Cohort 2. Change to add-on therapy occurred in most subjects during the first 12 months of analysis in both cohorts: 69.0% (*n* = 20) in Cohort 1-1 (July 2011–June 2012) and 67.3% (*n* = 72) in Cohort 2-2 (July 2014–June 2015).

In total, 335 subjects (8.38%) switched AD medication: 169 (8.5%) in Cohort 1 and 166 (8.3%) in Cohort 2. In Cohort 1, the most common reason for switching drugs was lack of effectiveness (*n* = 120; 6.0%), followed by adverse effects (*n* = 38; 1.9%), other (*n* = 10; 0.5%) and economic burden (*n* = 1; 0.1%). In Cohort 2, reasons for switching were lack of effectiveness (*n* = 103; 5.2%), adverse effects (*n* = 49; 2.5%), and other (*n* = 14; 0.7%). Differences between cohorts regarding reasons for switching medication were not statistically significant (*P* = 0.1866).

In subgroup analysis of Cohort 1, most subjects switched medications due to lack of effectiveness (*n* = 119) during the first year (Cohort 1-1, 57.1%), compared with the second (Cohort 1-2; 33.6%), and third year of study (Cohort 1-3; 9.2%); and rates for switching due to adverse effects (*n* = 38) in Cohorts 1-1, 1-2 and 1-3 were 63.2%, 10.5% and 26.3%, respectively. In subgroup analysis of Cohort 2, rates of subjects switching medications due to lack of effectiveness (*n* = 102) in Cohorts 2-1, 2-2 and 2-3 were 38.2%, 39.2% and 22.6%, respectively; and for those switching due to adverse effects (*n* = 49) were 49.0%, 24.5% and 26.5%, respectively.

**Fig. 2:**
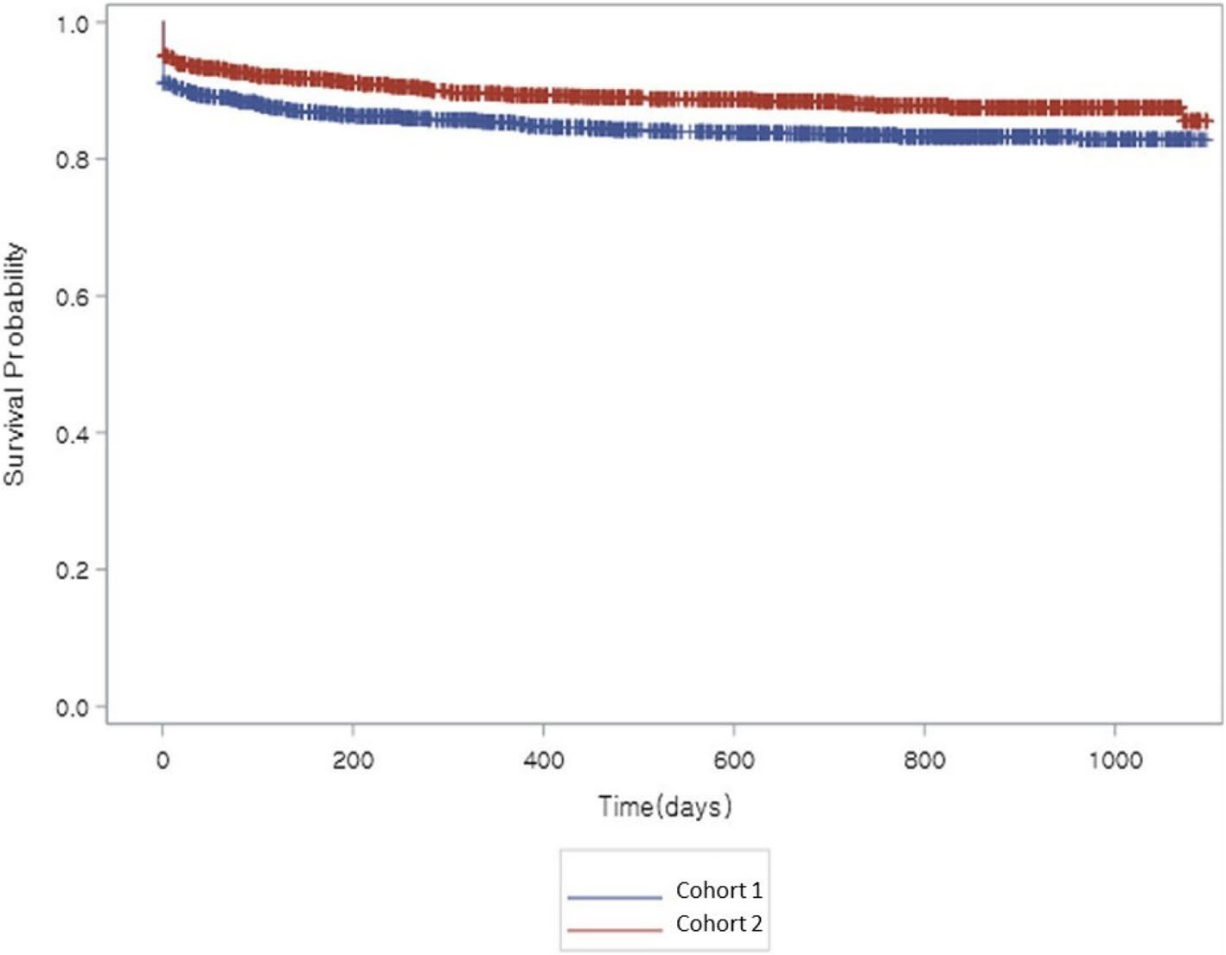
Kaplan-Meier analysis of time from initial Alzheimer’s disease treatment to diagnosis of depression or antidepressant prescription

Overall, mean ± SD time from initial AD treatment to diagnosis of depression or antidepressant prescription was 517.0 ± 350.4 days (*n* = 3,222). Kaplan-Meier analysis of time from initial AD treatment to diagnosis of depression or antidepressant prescription in Cohorts 1 and 2 is shown in Fig. 2. Mean ± SD time to depression diagnosis/antidepressant prescription was significantly prolonged in Cohort 2 (*n* = 1,586) compared with Cohort 1 (*n* = 1,636): 530.8 ± 352.6 *vs* 503.6 ± 347.9 days (Log-rank test *P* < 0.0001). In subjects stratified by disease severity at baseline, Cohort 2 prolongation of time to depression diagnosis/antidepressant prescription was found in the mild (*P* = 0.0001) and moderate (*P* = 0.0209) AD subgroups (Supplementary Table 2).

In patients who did not have a diagnosis of depression at baseline, time from initial treatment of AD to diagnosis of depression or antidepressant prescription for each medication is shown in Table 5. Mean time to diagnosis of depression or antidepressant prescription was significantly longer in Cohort 2 *vs* Cohort 1 for donepezil (521.9 *vs* 520.7 days; *P* = 0.0026) and rivastigmine (678.7 *vs* 505.8 days; *P* = 0.0220).

**Table 5:** Time from initial treatment (days) of Alzheimer's disease to diagnosis of depression or antidepressant prescription

		**Cohort 1**	**Cohort 2**	**Total**	*P* value*: Cohort 1 *vs* 2
Donepezil	n	1,406	1,335	2,741	
	Mean ± SD	520.7 ± 343.7	521.9 ± 353.8	521.3 ± 348.6	0.0026
Rivastigmine	n	247	162	409	
	Mean ± SD	505.8 ± 368.7	678.7 ± 316.4	574.3 ± 358.6	0.0220
Galantamine	n	134	213	347	
	Mean ± SD	433.2 ± 353.1	460.4 ± 314.0	449.9 ± 329.4	0.1065
Memantine	n	71	73	144	
	Mean ± SD	380.8 ± 314.6	449.5 ± 348.1	415.6 ± 332.6	0.3086
Combination donepezil + memantine	n		68	68	
	Mean ± SD	—	723.7 ± 366.7	723.7 ± 366.7	—

^*^Log-rank test. Mean (± SD**)** time from initial treatment to diagnosis of depression or antidepressant prescription is shown

## Discussion

This retrospective cohort study investigated changes in treatment patterns for subjects with newly diagnosed AD in Korea during two consecutive 3-year periods which were before (Cohort 1) and after (Cohort 2) the July 2014 revision of the national LTCI system regarding eligibility for dementia patients.

At baseline, a higher proportion of patients in Cohort 2 (July 2014 – June 2017) than Cohort 1 (July 2011 – June 2014) had one or more comorbidities which may reflect increased diagnosis and treatment of dementia in clinics visited for evaluation of non-dementia conditions. Depression was more commonly diagnosed in Cohort 2 which may reflect increased recognition of cognitive disturbances associated with depressive symptoms [15]. In contrast, stroke was less common in Cohort 2 and the reasons for this are unclear. Although stroke mortality in Korea has steadily decreased from 2010 to 2019 (by 12.8% from 2014 to 2019) due to better management of risk factors and improved medical interventions, the absolute number of incident strokes increased by 29.7% from 2014 to 2019 [16]. Moreover, based on an analysis of health insurance big data, the female incidence of stroke has decreased in Korea [17] and, as the AD patient population in our study was predominantly female, this may account for the observed decrease in stroke between Cohort 1 and Cohort 2.

Mean MMSE scores at the time of AD diagnosis or start of initial treatment were not significantly different between cohorts but, as there were significant differences in mean age and age category, then it is possible that age-related MMSE scores may differ for some age groups. These analyses remain to be done. Similarly, no significant difference was found in 1-year MMSE scores between cohorts and a “trend” of statistical significance was observed for change in MMSE scores from treatment start to 1 year’s end of treatment.

Initial medications administered to AD patients differed significantly between cohorts, and donepezil was most frequently administered – to more than three-quarters of patients. Combination AChEI + memantine was only given to Cohort 2 patients as insurance coverage for combination therapy was only available from October 2014. Recent results from the Observational Medical Outcome Partnership Common Data Model (OMOP CDM) which analyzed data from five hospitals in Korea during 2009-2019 also found that donepezil was the most prescribed anti-dementia medication (48.8%) among patients with newly diagnosed AD (*n* = 8,653), followed by memantine (18.1%), rivastigmine (9.0%), and galantamine (5.7%) [18].

Low medication persistence and/or adherence represents a significant challenge in treating patients with chronic diseases, including those with dementia [19, 20]. Medication persistence rates in the present study for donepezil, galantamine, rivastigmine and memantine were all high (≥98%). For comparison, the OMOP CDM study reported 12-month persistence rates of approximately 50% for donepezil and memantine and around 40% for rivastigmine and galantamine [18]. Differences in persistence rates may be due to differences in definitions of persistence and in study populations. Although mean medication persistence in our study was statistically higher in Cohort 1 *vs* 2 for donepezil and memantine, this was not clinically meaningful. Data indicate that several factors may influence persistence with dementia pharmacotherapy, including patient age, sex, ethnic/racial background, socioeconomic status, and region-specific reimbursement criteria, in addition to the extent and quality of interactions among patients, caregivers, and providers [20].

Depressive symptoms are common in AD, occurring in approximately 15% of patients [21]. Mean time to depression diagnosis/antidepressant prescription was significantly prolonged in Cohort 2 compared with Cohort 1. The prescription of depressive drugs other than those issued by psychiatry departments was more tightly regulated in earlier years which may have contributed to these results. In addition, prescriptions were checked only in EMRs from neurology departments. Mean time to diagnosis of depression or antidepressant prescription was significantly longer for donepezil (by approximately 1 day) and rivastigmine (by nearly 173 days).

The mean time from AD diagnosis to the start of initial therapy was slightly longer (by approximately 1 day) in Cohort 2 compared with Cohort 1. This may be due to a strain on AD diagnostic facilities due to increased patient numbers. However, in patients who discontinued or changed their initial treatment, the mean duration of treatment was significantly longer in Cohort 2 (by 49 days). This likely reflects the change in LTCI policy which enabled increased access to long-term care for patients. Introduction of the national LTCI-funded cognitive function training program was also associated with a significant reduction in the decline of cognitive function in older people with mild dementia after, compared to before, its introduction [11].

The proportion of patients who discontinued or changed their initial treatment was also significantly lower in Cohort 2 and appear to be associated with the policy revision in 2014. Lack of effectiveness and adverse effects were the main reasons for discontinuing or changing treatment, but as many subjects (*n* = 1,587; 39.7% of all subjects) were lost to follow-up, differences between cohorts were limited by relatively low numbers of patients. Predictors of discontinuation or change in therapy was beyond the scope of this study. However, a 2-year European prospective cohort study of patients with mild-to-moderate AD initiating AChEIs (*n* = 557) reported that predictors of discontinuation were behavioral disturbances, decline in MMSE score, AD-related hospitalization, low body mass index (BMI) and falls; and predictors of switching treatment were MMSE score, decline in activities of daily living score, shorter AD duration, aberrant motor behavior, and higher nurse resource use [22].

The main limitations of the current study reflect those associated with the retrospective nature of the study design which analyzed data from EMRs. Data pre-processing and data quality (e.g. incomplete, inaccurate and/or missing data) challenges, and the potential for limited generalizability, are recognized challenges encountered when using EMR data for secondary research purposes [23]. For example, this may have impacted findings relating to medication persistence because it was not possible to differentiate between patients who actually took the medication and those who did not. There may also be differences in patient care between hospitals such as neuropsychological examinations, interval between examinations etc. although all patients were treated by neurologists. Antidepressants are often prescribed by psychiatrists due to insurance regulations, and this may have also led to differences in care of patients between hospitals. As there were a number of policy changes over several years, only large changes to policy were considered. Finally, as the primary aim of the study was to examine change in treatment patterns between cohorts, in depth statistical analyses such as Cox regression to account for confounding factors for differences in MMSE were not performed. However, we are planning more detailed *post-hoc* analyses (including Cox regression) for a subsequent publication.

## Conclusions

This study compared cohorts before and after revision of the national LTCI system for dementia patients in Korea and found no significant difference between cohorts in mean MMSE scores at the time of AD diagnosis or start of initial treatment. The reduction in the proportion of patients who discontinued or changed their initial treatment, and the significant increase in mean duration of treatment, were observed following revision of the LTCI policy including national dementia management, which enabled increased access to long-term care for patients with dementia and positive effects on care of depression. Large-scale research projects including long-term prospective studies are needed to continue to monitor the care of dementia patients in Korea.

### Supplementary Information

**Additional file 1: Supplementary Table 1.** List of study sites and corresponding Institutional Review Boards (IRB) that reviewed and approved the study protocol.

**Additional file 2: Supplementary Table 2.** Change in MMSE, treatment duration, discontinuation/changing treatment, medication persistence and time to diagnosis of depression/ prescription of antidepressants by severity of Alzheimer's disease (AD) at baseline.

**Additional file 3: Supplementary Table 3.** Initial treatment medication in Cohort subgroups (analyzed by 12-month periods).

**Additional file 4: Supplementary Table 4.** Reasons for discontinuation/interruption of initial treatment in Cohort subgroups (analyzed by 12-month periods).

## Data Availability

The datasets generated and/or analyzed during the current study are not publicly available due to ethical constraints but are available from the corresponding author on reasonable request.

## References

[1] Alzheimer's Disease International. Dementia statistics. 2022. Available from: https://www.alzint.org/about/dementia-facts-figures/dementia-statistics/ ( Accessed 16 August 2022).

[2] Jang JW, Park JH, Kim S, Lee SH, Lee SH, Kim YJ (2021). Prevalence and Incidence of Dementia in South Korea: A Nationwide Analysis of the National Health Insurance Service Senior Cohort. J Clin Neurol.

[3] Choi YJ, Kim S, Hwang YJ, Kim C (2021). Prevalence of Dementia in Korea Based on Hospital Utilization Data from 2008 to 2016. Yonsei Med J.

[4] Lee M-S (2019). Preparation and Measures for Elderly with Dementia in Korea: Focus on National Strategies and Action Plan against Dementia. J Agric Med Community Health.

[5] Shin J-H (2022). Dementia Epidemiology Fact Sheet 2022. Ann Rehabil Med.

[6] Shon C, Yoon H (2021). Health-economic burden of dementia in South Korea. BMC Geriatr.

[7] National Institute of Dementia. Korean Dementia Observatory 2022. Seoul: National Institute of Dementia; 2023. Available from: https://www.nid.or.kr/info/dataroom_view.aspx?bid=257 . Accessed 9 November 2023.

[8] Lee DW, Seong S-J (2018). Korean national dementia plans: from 1st to 3^rd^. J Korean Med Assoc.

[9] Hampel H, Vergallo A, Iwatsubo T, Cho M, Kurokawa K, Wang H, Kurzman HR, Chen C (2022). Evaluation of major national dementia policies and health-care system preparedness for early medical action and implementation. Alzheimers Dement.

[10] Jeon B, Kwon S (2017). Health and Long-Term Care Systems for Older People in the Republic of Korea: Policy Challenges and Lessons. Health Syst Reform.

[11] Ju YJ, Nam CM, Lee SG, Park S, Hahm MI, Park EC (2019). Evaluation of the South Korean national long-term care insurance-funded cognitive function training programme for older people with mild dementia. Age Ageing..

[12] Tombaugh TN, McIntyre NJ (1992). The mini-mental state examination: a comprehensive review. J Am Geriatr Soc..

[13] Morris JC (1997). Clinical dementia rating: a reliable and valid diagnostic and staging measure for dementia of the Alzheimer type. Int Psychogeriatr.

[14] Reisberg B, Ferris SH, de Leon MJ, Crook T (1982). The Global Deterioration Scale for assessment of primary degenerative dementia. Am J Psychiatry.

[15] Padovani A, Antonini A, Barone P, Bellelli G, Fagiolini A, Ferini Strambi L, Sorbi S, Stocchi F (2023). Exploring depression in Alzheimer's disease: an Italian Delphi Consensus on phenomenology, diagnosis, and management. Neurol Sci.

[16] Jung SH (2022). Stroke Rehabilitation Fact Sheet in Korea. Ann Rehabil Med.

[17] Korean Ministry of Health and Welfare (MOHW). Press release (3 April 2017). 4 out of 5 “stroke” patients are over 60. Available from http://www.mohw.go.kr/react/al/sal0301vw.jsp?PAR_MENU_ID=04&MENU_ID=0403&CONT_SEQ=339009&page=1 . Accessed 14 Nov 2022.

[18] Byun J, Lee DY, Jeong CW, Kim Y, Rhee HY, Moon KW, Heo J, Hong Y, Kim WJ, Nam SJ, Choi HS, Park JI, Chun IK, Bak SH, Lee K, Byeon GH, Kim KL, Kim JA, Park YJ, Kim JH, Lee EJ, Lee SA, Kwon SO, Park SW, Kasani PH, Kim JK, Kim Y, Kim S, Jang JW (2022). Analysis of treatment pattern of anti-dementia medications in newly diagnosed Alzheimer's dementia using OMOP CDM. Sci Rep.

[19] Nieuwlaat R, Wilczynski N, Navarro T, Hobson N, Jeffery R, Keepanasseril A, Agoritsas T, Mistry N, Iorio A, Jack S, Sivaramalingam B, Iserman E, Mustafa RA, Jedraszewski D, Cotoi C, Haynes RB. Interventions for enhancing medication adherence. Cochrane Database Syst Rev. 2014;2014(11):CD000011. 10.1002/14651858.CD000011.pub4 PMC7263418 25412402

[20] Maxwell CJ, Stock K, Seitz D, Herrmann N (2014). Persistence and adherence with dementia pharmacotherapy: relevance of patient, provider, and system factors. Can J Psychiatry.

[21] Asmer MS, Kirkham J, Newton H, Ismail Z, Elbayoumi H, Leung RH, Seitz DP. Meta-Analysis of the Prevalence of Major Depressive Disorder Among Older Adults with Dementia. J Clin Psychiatry. 2018;79(5):17r11772. 10.4088/JCP.17r11772 30085437

[22] Gardette V, Lapeyre-Mestre M, Piau A, Gallini A, Cantet C, Montastruc JL, Vellas B, Andrieu S; ICTUS Group. A 2-year prospective cohort study of antidementia drug non-persistency in mild-to-moderate Alzheimer's disease in Europe: predictors of discontinuation and switch in the ICTUS study. CNS Drugs. 2014;28(2):157–170. 10.1007/s40263-013-0133-3 24408842

[23] Edmondson ME, Reimer AP (2020). Challenges frequently encountered in the secondary use of electronic medical record data for research. Comput Inform Nurs.

