# Supplementary material for: Changes in dementia treatment patterns associated with changes in the National Policy in South Korea among patients with newly diagnosed Alzheimer’s disease between 2011 and 2017: results from the multicenter, retrospective CAPTAIN study
Source: BMC Public Health. 2024 Jan 12;24:168. doi: 10.1186/s12889-024-17671-2 (PMC10787419; doi:10.1186/s12889-024-17671-2)
Supplement: Supplementary file 1 — Additional file 1: Supplementary Table 1. List of study sites and corresponding Institutional Review Boards (IRB) that reviewed and approved the study protocol. [file 12889_2024_17671_MOESM1_ESM.docx]

**Supplementary Table 1. List of study sites and corresponding Institutional Review Boards (IRB) that reviewed and approved the study protocol.**

| **No** | **Site** | **IRB Name** |
| --- | --- | --- |
| 1 | Sungae Hospital | Sungae Hospital IRB |
| 2 | Gwangju Veterans Hospital | Gwangju Veterans Hospital IRB |
| 3 | Namwon Medical Center | The Public Institutional Review Board (Public IRB)^a^ |
| 4 | Daegu Fatima Hospital | Daegu Fatima Hospital IRB |
| 5 | Daejeon St. Mary's Hospital | Daejeon St. Mary's Hospital IRB |
| 6 | Baekje General Hospital | The Public Institutional Review Board (Public IRB)^a^ |
| 7 | Seoul Metropolitan Government-Seoul National University Boramae Medical Center | Seoul Metropolitan Government-Seoul National University Boramae Medical Center IRB |
| 8 | Busan St. Mary's Hospital | Busan St. Mary's Hospital IRB |
| 9 | Andong Medical Group Hospital | Andong Medical Group Hospital IRB |
| 10 | Yeosu Chonnam Hospital | The Public Institutional Review Board (Public IRB)^a^ |
| 11 | Jeju National University Hospital | Jeju National University Hospital IRB |
| 12 | Veterans Healthcare Medical Center | Veterans Healthcare Medical Center IRB |
| 13 | Chamjoeun Hospital | The Public Institutional Review Board (Public IRB)^a^ |
| 14 | Changwon Fatima Hospital | Changwon Fatima Hospital IRB |
| 15 | Hallym Hospital | The Public Institutional Review Board (Public IRB)^a^ |
| 16 | National Health Insurance Service Ilsan Hospital | National Health Insurance Service Ilsan Hospital IRB |
| 17 | BongSeng Memorial Hospital | BongSeng Memorial Hospital IRB |

^a^ Study site does not have its own IRB; study review and approval was obtained from the Public Institutional Review Board (Public IRB).
