# Supplementary material for: Changes in dementia treatment patterns associated with changes in the National Policy in South Korea among patients with newly diagnosed Alzheimer’s disease between 2011 and 2017: results from the multicenter, retrospective CAPTAIN study
Source: BMC Public Health. 2024 Jan 12;24:168. doi: 10.1186/s12889-024-17671-2 (PMC10787419; doi:10.1186/s12889-024-17671-2)
Supplement: Supplementary file 2 — Additional file 2: Supplementary Table 2. Change in MMSE, treatment duration, discontinuation/changing treatment, medication persistence and time to diagnosis of depression/ prescription of antidepressants by severity of Alzheimer's disease (AD) at baseline. [file 12889_2024_17671_MOESM2_ESM.docx]

**Supplementary Table 2. Change in MMSE, treatment duration, discontinuation/changing treatment, medication persistence and time to diagnosis of depression/ prescription of antidepressants by severity of Alzheimer's disease (AD) at baseline.**

|  |  |  | **Cohort 1**  **(n = 1,998)** | **Cohort 2**  **(n = 1,999)** | **Total**  **(N = 3,997)** | ***P* value: Cohort 1 *vs* 2** |
| --- | --- | --- | --- | --- | --- | --- |
| MMSE score at treatment start (baseline) | | | | | | |
| Normal | | Subjects, n (%) | 38 (80.9) | 9 (19.2) | 47 (100.0) |  |
|  |  | Mean ± SD | 27.8 ± 0.8 | 27.3 ± 0.5 | 27.7 ± 0.7 | 0.0672 ^a^ |
| Mild | | Subjects, n (%) | 583 (47.9) | 634 (52.1) | 1,217 (100.0) |  |
|  |  | Mean ± SD | 23.5 ± 1.7 | 23.3 ± 1.7 | 23.4 ± 1.7 | 0.2058 ^a^ |
| Moderate | | Subjects, n (%) | 1,133 (49.6) | 1,151 (50.4) | 2,284 (100.0) |  |
|  |  | Mean ± SD | 15.5 ± 3.0 | 15.6 ± 3.0 | 15.5 ± 3.0 | 0.5094 ^a^ |
| Severe | | Subjects, n (%) | 244 (54.3) | 205 (45.7) | 449 (100.0) |  |
|  |  | Mean ± SD | 6.0 ± 2.8 | 6.0 ± 2.8 | 6.0 ± 2.8 | 0.9274 ^b^ |
| Change in MMSE score from treatment start to end of 1 year's treatment | | | | | | |
| Normal | | Subjects, n (%) | 6 (60.0) | 4 (40.0) | 10 (100.0) |  |
|  |  | Mean ± SD | -2.67 ± 2.66 | -2.00 ± 2.16 | -2.40 ± 2.37 | 0.6558 ^a^ |
| Mild | | Subjects, n (%) | 205 (45.6) | 245 (54.4) | 450 (100.0) |  |
|  |  | Mean ± SD | -0.40 ± 2.73 | -1.26 ± 3.11 | -0.87 ± 2.97 | 0.0021 ^c^ |
| Moderate | | Subjects, n (%) | 317 (44.0) | 403 (56.0) | 720 (100.0) |  |
|  |  | Mean ± SD | 0.34 ± 3.91 | 0.19 ± 3.39 | 0.25 ± 3.63 | 0.5852 ^c^ |
| Severe | | Subjects, n (%) | 60 (52.2) | 55 (47.8) | 115 (100.0) |  |
|  |  | Mean ± SD | 1.85 ± 3.91 | 2.38 ± 4.87 | 2.10 ± 4.38 | 0.8726 ^a^ |
| Time from diagnosis of AD to start of initial treatment (days) | | | | | | |
| Normal | | Subjects, n (%) | 38 (80.9) | 9 (19.2) | 47 (100.0) |  |
|  |  | Mean ± SD | 19.2 ± 58.9 | 51.8 ± 111.5 | 25.5 ± 71.6 | 0.122 ^c^ |
| Mild | | Subjects, n (%) | 583 (47.9) | 634 (52.1) | 1,217 (100.0) |  |
|  |  | Mean ± SD | 9.6 ± 52.8 | 9.9 ± 44.9 | 9.8 ± 44.8 | 0.0427 ^c^ |
| Moderate | | Subjects, n (%) | 1,133 (49.6) | 1,151 (50.4) | 2,284 (100.0) |  |
|  |  | Mean ± SD | 6.8 ± 36.2 | 8.1 ± 34.0 | 7.5 ± 35.1 | 0.0034 ^c^ |
| Severe | | Subjects, n (%) | 244 (54.3) | 205 (45.7) | 449 (100.0) |  |
|  |  | Mean ± SD | 6.5 ± 21.7 | 7.2 ± 31.5 | 6.8 ± 26.6 | 0.9021 ^c^ |
| Number and proportion of subjects discontinuing or changing treatment | | | | | | |
| Normal | | Subjects, n (%) | 38 (80.9) | 9 (19.2) | 47 (100.0) |  |
|  |  | n (%) | 17 (44.7) | 4 (44.4) | 21 (44.7) | 1.000 ^d^ |
| Mild | | Subjects, n (%) | 583 (47.9) | 634 (52.1) | 1,217 (100.0) |  |
|  |  | n (%) | 299 (51.3) | 293 (46.2) | 592 (48.6) | 0.0770 ^e^ |
| Moderate | | Subjects, n (%) | 1,133 (49.6) | 1,151 (50.4) | 2,284 (100.0) |  |
|  |  | n (%) | 678 (59.8) | 570 (49.5) | 1,248 (54.6) | <0.0001 ^e^ |
| Severe | | Subjects, n (%) | 244 (54.3) | 205 (45.7) | 449 (100.0) |  |
|  |  | n (%) | 165 (67.6) | 127 (62.0) | 292 (65.0) | 0.2093 ^e^ |
| Initial treatment period (days) for subjects who discontinued or changed initial treatment for AD | | | | | | |
| Normal | | Subjects, n (%) | 38 (80.9) | 9 (19.2) | 47 (100.0) |  |
|  |  | Mean ± SD | 430.6 ± 329.9 | 466.4 ± 306.7 | 437.5 ± 322.6 | 0.9677 ^c^ |
| Mild | | Subjects, n (%) | 583 (47.9) | 634 (52.1) | 1,217 (100.0) |  |
|  |  | Mean ± SD | 336.8 ± 318.2 | 372.5 ± 320.0 | 355.4 ± 319.5 | 0.0317 ^c^ |
| Moderate | | Subjects, n (%) | 1,133 (49.6) | 1,151 (50.4) | 2,284 (100.0) |  |
|  |  | Mean ± SD | 291.5 ± 313.6 | 342.7 ± 311.8 | 317.3 ± 313.7 | <0.0001 ^c^ |
| Severe | | Subjects, n (%) | 244 (54.3) | 205 (45.7) | 449 (100.0) |  |
|  |  | Mean ± SD | 233.0 ± 268.0 | 309.5 ± 324.1 | 267.9 ± 297.1 | 0.0286 ^c^ |
| Medication persistence rate (%) for cholinesterase inhibitors (ChEIs) or memantine | | | | | | |
| Normal | Donepezil | Subjects, n (%) | 38 (80.9) | 9 (19.2) | 47 (100.0) |  |
|  |  | Mean ± SD | 97.4 ± 14.5 | 91.7 ± 25.0 | 96.2 ± 17.1 | 0.5239 ^b^ |
|  | Galantamine | Subjects, n (%) | 1 (100.0) | 0 | 1 (100.0) |  |
|  |  | Mean ± SD | 100.0 | – | 100.0 | – |
|  | Rivastigmine | Subjects, n (%) | 4 (100.0) | 0 | 4 (100.0) |  |
|  |  | Mean ± SD | 100.0 ± 0.0 | – | 100.0 ± 0.0 | – |
|  | Memantine | Subjects, n (%) | 0 | 0 | 0 |  |
|  |  | Mean ± SD | – | – | – | – |
| Mild | Donepezil | Subjects, n (%) | 406 (47.2) | 455 | 861 (100.0) |  |
|  |  | Mean ± SD | 99.4 ± 5.4 | 98.9 ± 6.1 | 99.1 ± 5.8 | 0.0737 ^a^ |
|  | Galantamine | Subjects, n (%) | 43 (38.1) | 70 (61.9) | 113 (100.0) |  |
|  |  | Mean ± SD | 97.6 ± 8.1 | 99.8 ± 1.3 | 98.9 ± 5.2 | 0.0285 ^a^ |
|  | Rivastigmine | Subjects, n (%) | 123 (59.1) | 85 (40.9) | 208 (100.0) |  |
|  |  | Mean ± SD | 99.0 ± 8.6 | 100.0 ± 0.0 | 99.4 ± 6.7 | 0.1495 ^a^ |
|  | Memantine | Subjects, n (%) | 3 (42.9) | 4 (57.1) | 7 (100.0) |  |
|  |  | Mean ± SD | 100.0 ± 0.0 | 100.0 ± 0.0 | 100.0 ± 0.0 | 1.0000 ^a^ |
| Moderate | Donepezil | Subjects, n (%) | 865 (50.9) | 835 (49.1) | 1,700 (100.0) |  |
|  |  | Mean ± SD | 98.5 ± 8.1 | 98.5 ± 6.3 | 98.5 ± 7.3 | 0.0023 ^a^ |
|  | Galantamine | Subjects, n (%) | 89 (38.5) | 142 (61.5) | 231 (100.0) |  |
|  |  | Mean ± SD | 99.2 ± 4.7 | 99.4 ± 4.6 | 99.3 ± 4.6 | 0.7173 ^a^ |
|  | Rivastigmine | Subjects, n (%) | 119 (57.5) | 88 (42.5) | 207 (100.0) |  |
|  |  | Mean ± SD | 97.5 ± 12.5 | 99.6 ± 4.0 | 98.4 ± 9.9 | 0.1368 ^a^ |
|  | Memantine | Subjects, n (%) | 42 (30.4) | 96 (69.6) | 138 (100.0) |  |
|  |  | Mean ± SD | 98.8 ± 7.7 | 98.7 ± 7.1 | 98.7 ± 7.3 | 0.0230 ^a^ |
| Severe | Donepezil | Subjects, n (%) | 210 (54.7) | 174 (45.3) | 384 (100.0) |  |
|  |  | Mean ± SD | 98.8 ± 4.9 | 92.2 ± 8.0 | 98.1 ± 6.5 | 0.0424 ^a^ |
|  | Galantamine | Subjects, n (%) | 3 (50.0) | 3 (50.0) | 6 (100.0) |  |
|  |  | Mean ± SD | 100.0 ± 0.0 | 100.0 ± 0.0 | 100.0 ± 0.0 | 1.0000 ^a^ |
|  | Rivastigmine | Subjects, n (%) | 3 (37.5) | 5 (62.5) | 8 (100.0) |  |
|  |  | Mean ± SD | 100.0 ± 0.0 | 100.0 ± 0.0 | 100.0 ± 0.0 | 1.0000 ^a^ |
|  | Memantine | Subjects, n (%) | 26 (38.2) | 42 (61.8) | 68 (100.0) |  |
|  |  | Mean ± SD | 98.6 ± 6.9 | 98.4 ± 6.5 | 98.5 ± 6.6 | 0.9182 ^b^ |
| Time from initial treatment (days) for AD to diagnosis of depression/ prescription of antidepressants | | | | | | |
| Normal | | Subjects, n (%) | 38 (82.6) | 8 (17.4) | 46 (100.0) |  |
|  |  | Mean ± SD | 550.3 ± 331.8 | 651.3 ± 204.2 | 567.9 ± 313.9 | 0.3176 ^c^ |
| Mild | | Subjects, n (%) | 552 (48.5) | 585 (51.5) | 1,137 (100.0) |  |
|  |  | Mean ± SD | 481.0 ± 352.5 | 534.4 ± 350.2 | 508.5 ± 352.2 | 0.0001 ^c^ |
| Moderate | | Subjects, n (%) | 1,048 (49.6) | 1,067 (50.4) | 2,115 (100.0) |  |
|  |  | Mean ± SD | 510.4 ± 344.5 | 521.6 ± 350.7 | 516.0 ± 347.6 | 0.0209 ^c^ |
| Severe | | Subjects, n (%) | 220 (53.5) | 191 (46.5) | 411 (100.0) |  |
|  |  | Mean ± SD | 549.2 ± 353.9 | 588.1 ± 366.9 | 567.3 ± 360.1 | 0.4862 ^c^ |

^a^ Wilcoxon rank sum test; ^b^ Independent t-test; ^c^ Log-rank test; ^d^ Fisher’s exact test; ^e^ Chi square test
