# Supplementary material for: Changes in dementia treatment patterns associated with changes in the National Policy in South Korea among patients with newly diagnosed Alzheimer’s disease between 2011 and 2017: results from the multicenter, retrospective CAPTAIN study
Source: BMC Public Health. 2024 Jan 12;24:168. doi: 10.1186/s12889-024-17671-2 (PMC10787419; doi:10.1186/s12889-024-17671-2)
Supplement: Supplementary file 3 — Additional file 3: Supplementary Table 3. Initial treatment medication in Cohort subgroups (analyzed by 12-month periods). [file 12889_2024_17671_MOESM3_ESM.docx]

**Supplementary Table 3. Initial treatment medication in Cohort subgroups (analyzed by 12-month periods)**

|  | Cohort 1-1  (n = 897) | Cohort 1-2  (n = 556) | Cohort 1-3  (n = 545) | Cohort 2-1  (n = 865) | Cohort 2-2  (n = 582) | Cohort 2-3  (n = 552) |
| --- | --- | --- | --- | --- | --- | --- |
|  | n (%) | | | | | |
| Donepezil | 706 (78.7) | 420 (75.5) | 415 (76.2) | 617 (71.3) | 409 (70.3) | 431 (78.1) |
| Rivastigmine | 129 (14.4) | 58 (10.4) | 63 (11.6) | 107 (12.4) | 59 (10.1) | 14 (2.5) |
| Galantamine | 45 (5.0) | 56 (10.1) | 35 (6.4) | 61 (7.1) | 89 (15.3) | 68 (12.3) |
| Memantine | 17 (1.9) | 22 (4.0) | 32 (5.9) | 27 (3.1) | 21 (3.6) | 28 (5.1) |
| Combination donepezil + memantine | 0 (0.0) | 0 (0.0) | 0 (0.0) | 53 (6.1) | 4 (0.7) | 11 (2.0) |
| Total | 897 (100.0) | 556 (100.0) | 545 (100.0) | 865 (100.0) | 582 (100.0) | 552 (100.0) |
| *P* value* | – | <0.0001 | 0.0777 | <0.0001 | <0.0001 | <0.0001 |

Percentages shown are for the proportion of subjects in each subcohort. * Chi-square test

Analysis period of sub-groups:

Cohort 1-1: July 2011–June 2012; Cohort 1-2: July 2012– June 2013; Cohort 1-3: July 2013–June 2014; Cohort 2-1: July 2014–June 2015; Cohort 2-2: July 2015–June 2016; Cohort 2-3: July 2016–June 2017
