# Supplementary material for: Changes in dementia treatment patterns associated with changes in the National Policy in South Korea among patients with newly diagnosed Alzheimer’s disease between 2011 and 2017: results from the multicenter, retrospective CAPTAIN study
Source: BMC Public Health. 2024 Jan 12;24:168. doi: 10.1186/s12889-024-17671-2 (PMC10787419; doi:10.1186/s12889-024-17671-2)
Supplement: Supplementary file 4 — Additional file 4: Supplementary Table 4. Reasons for discontinuation/interruption of initial treatment in Cohort subgroups (analyzed by 12-month periods). [file 12889_2024_17671_MOESM4_ESM.docx]

**Supplementary Table 4. Reasons for discontinuation/interruption of initial treatment in Cohort subgroups (analyzed by 12-month periods)**

|  | **Cohort 1-1**  **(n = 897)** | **Cohort 1-2**  **(n = 556)** | **Cohort 1-3**  **(n = 545)** | **Cohort 2-1 (n = 865)** | **Cohort 2-2 (n = 582)** | **Cohort 2-3 (n = 552)** |
| --- | --- | --- | --- | --- | --- | --- |
|  | n (%) | | | | | |
| Lost to follow-up | 478 (53.3) | 245 (44.1) | 178 (32.7) | 366 (42.3) | 199 (34.2) | 86 (15.6) |
| Lack of effectiveness | 7 (0.8) | 3 (0.5) | 2 (0.4) | 8 (0.9) | 3 (0.5) | 1 (0.2) |
| Adverse effects | 4 (0.5) | 1 (0.2) | 2 (0.4) | 3 (0.4) | 9 (1.6) | 2 (0.4) |
| Death | 5 (0.6) | 1 (0.2) | 0 (0.0) | 6 (0.7) | 6 (1.03) | 0 (0.00) |
| Economic burden | 0 (0.0) | 0 (0.0) | 0 (0.0) | 1 (0.1) | 0 (0.0) | 1 (0.2) |
| Symptom improvement | 0 (0.0) | 1 (0.2) | 0 (0.0) | 0 (0.0) | 0 (0.0) | 0 (0.00) |
| Other | 11 (2.2) | 7 (2.7) | 16 (8.1) | 22 (5.4) | 5 (2.3) | 3 (3.2) |
| Total | 505 | 258 | 198 | 406 | 222 | 93 |
| *P* value* | – | 0.7373 | 0.0504 | 0.3238 | 0.0142 | 0.3355 |

Percentages shown are for the proportion of subjects in each subcohort

* Fisher's exact test

Analysis period of sub-groups:

Cohort 1-1: July 2011–June 2012; Cohort 1-2: July 2012–June 2013; Cohort 1-3: July 2013–June 2014; Cohort 2-1: July 2014–June 2015; Cohort 2-2: July 2015–June 2016; Cohort 2-3: July 2016–June 2017
